# Nifedipine in Congenital Hyperinsulinism- A Case Report

**DOI:** 10.4274/jcrpe.1978

**Published:** 2015-06-03

**Authors:** Papiya Khawash, Khalid Hussain, Sarah E. Flanagan, Sudip Chatterjee, Dhananjoy Basak

**Affiliations:** 1 Park Clinic, Clinic of Paediatrics and Neonatology, Kolkata, India; 2 Institute of Child Health, University College, Developmental Endocrinology Research Group, Clinical and Molecular Genetics Unit, London, UK; 3 University of Exeter Faculty of Medicine, Institute Biomedical and Clinical Science, Molecular Genetics, Exeter, UK

**Keywords:** congenital hyperinsulinism, Nifedipine, octreotide, continuous glucose monitoring sensor

## Abstract

Congenital hyperinsulinism (CHI) is the commonest cause of persistent hypoglycemia in neonates. Diazoxide is the first-line drug in its treatment, but the more severe cases are usually diazoxide-resistant. Recessive ABCC8 and KCNJ11 mutations are responsible for most (82%) of the severe diazoxide-unresponsive CHI. Oral nifedipine has been effective in isolated cases of CHI. Successful treatment of diazoxide-unresponsive CHI with a combination of octreotide and nifedipine has been reported in a single isolated case so far. We report here a case of diazoxide-resistant CHI due to homozygous ABCC8 nonsense mutation. In this case, hypoglycaemia uncontrolled by pancreatectomy and octreotide alone showed a good response to a combination of nifedipine and octreotide. Octreotide was tapered off by one year age and thereafter the child is euglycaemic on oral nifedipine alone. Continuous glucose monitoring sensor was used as an aid to monitor glycaemic control and was found to be a safe and reliable option reducing the number of needle-pricks in small children.

## INTRODUCTION

Congenital hyperinsulinism (CHI) comprises a heterogeneous group of genetic disorders with the common finding of recurrent episodes of hyperinsulinemic hypoglycemia due to an inappropriate secretion of insulin by the pancreatic β-cells. It is the commonest cause of persistent hypoglycemia in the neonatal and infancy periods. The incidence is estimated at 1/50000 live births, but may be as high as 1/2500 in countries where consanguinity is common (1). Mutations in the ABCC8 and KCNJ11 genes, which encode subunits of the ATP-sensitive potassium channel (KATP) in the pancreatic β-cell, are identified in approximately 40 to 45% of these patients and are responsible for most (82%) of the severe diazoxide-unresponsive CHI. Mutations have been identified in seven other genes in approximately 5 to 10% of the cases, while the genetic etiology of the rest is still unknown. In western countries, 45-60% of diazoxide-unresponsive CHI are focal forms, whereas 40-45% are diffuse forms (1,2,3).

In CHI, a high-glucose infusion rate is required to prevent hypoglycemia. The hypoglycemia responds to exogenous glucagon, since glucagon stimulates glycogen breakdown and gluconeogenesis. As insulin inhibits lipolysis, there is no compensatory generation of ketone bodies which could serve as an alternative fuel for the brain to protect it from hypoglycemia (1). Rapid diagnosis and prompt management of the hypoglycaemia are vital in preventing brain damage and mental retardation (4). The severity of CHI may be evaluated by the rate of glucose infusion required to maintain normoglycemia (1), though this may not necessarily be always true.

The first-line drug in the treatment of CHI is diazoxide (1,5). However, it is generally ineffective for the most severe, neonatal-onset, recessive and focal forms of KATP-CHI (5). Octreotide and glucagon can be used in patients who show no response to diazoxide. Calcium-channel blockers, like nifedipine, showed a clear efficiency in mouse models (6) and in isolated case reports (7,8,9,10). While most HI centers did not observe any response to nifedipine in large series of patients (1), it has been shown to be an effective treatment in a small number of patients with diazoxide-unresponsive CHI (7,8,9,10). The literature regarding the use of nifedipine in CHI is limited and conflicting and there is controversy about the dosage, side-effects and efficacy of short- versus long-acting formulations.

Here we report a case of a baby girl with diazoxide-unresponsive CHI treated with subcutaneous (S/c) octreotide and oral nifedipine for persistent hypoglycaemia after partial pancreatectomy. At 11/2 years, the patient is now on oral nifedipine only for past 6 months after octreotide was tapered off and stopped by 1 year of age. Genetic testing of the child showed that she is a compound heterozygote for an ABCC8 missense mutation. We also found continuous glucose monitoring sensor (CGMS) to be a safe, effective way of home monitoring in very small children and it reduced the number of needle-pricks for glucose-monitoring particularly when dosage of medications needed tapering or titration, in addition to instilling confidence in parents worried about asymptomatic hypoglycaemia regarding home management of the condition.

## CASE REPORT

An 11-day-old baby girl was referred with history of recurrent convulsions and severe, persistent hypoglycaemia. The baby was born of non-consanguineous marriage to a non-diabetic mother by elective Caesarean section at term. Baby was macrosomic (birth weight-4.7 kg) with no dysmorphic features. On admission, the patient needed a glucose infusion of 24 mg/kg/min via central line to maintain euglycaemia.

Investigations revealed inappropriately high levels of serum insulin and C-peptide levels during hypoglycemic episodes (glucose <40 mg/dL) [insulin - 66.4 μIU/mL (normal - 4 to 24.9) and C-peptide level - 9.8 ng/mL (normal - 1.1 to 3.6 ng/mL)]. Serum growth hormone, thyroid and cortisol levels were normal. A glucagon response was positive i.e. an increase of glucose level >50 mg/dL within 30 minutes of glucagon injection was recorded. Magnetic resonance imaging scan of the abdomen did not reveal any localized lesion in the pancreas. Based on these findings, a diagnosis of CHI was made and genetic testing of the child and both parents showed that she is a compound heterozygote for an ABCC8 missense mutation, p.R1419C (c.4255C>T) and a deletion of ABCC8 exon 3 (c.291-?_412+?del) thus confirming diffuse disease. Recommended medications were either not well-tolerated or difficult to procure. Surgery in the form of a limited subtotal pancreatectomy was discussed with the parents and performed.

Histopathological examination after the pancreatectomy confirmed the diagnosis of CHI due to diffuse lesion.

Post-operatively after an initial drop, glucose requirement gradually increased by day 4 to 20 mg/kg/min, only marginally less than pre-operative requirements. Oral diazoxide had not been tolerated causing severe, persistent vomiting. Octreotide was started S/cly at a dose of 5 µg/kg/d 8 hrly with doses being gradually increased to 30 µg/kg/d 6 hrly. By day 40, the baby was maintaining euglycaemia on full enteral 2 hrly feeds and S/c octreotide (30 µg/kg/day) without glucose infusion and was discharged to home.

**Figure 1:**
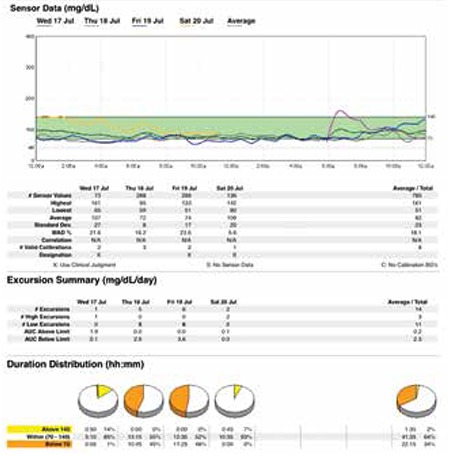
CGMS data showing euglycaemia (glucose >60mg/dl) after starting oral nifedipine along with s/c octreotide in post-pancreatectomy patient with CHI.

However, within 48 hours the baby was readmitted with feeding problems and episodes of symptomatic hypoglycaemia. In hospital, on reassessing the situation, we felt that in order to avoid dangerous pre-feed dips of blood glucose and early morning hypoglycaemia, a small but continuous infusion of iv glucose at the rate 1 to 1.5 mg/kg/min was still required. Calorie-densers (like coconut oil and human milk fortifiers) were added to feeds which though well-tolerated did not stop hypoglycaemic episodes. Re-surgery, i.e. sub-total pancreatectomy, was not considered as an option. A trial of oral nifedipine was given which was started at the lowest dose and gradually increased to 0.8 mg/kg/day over the next 6 days. This was well-tolerated with no side effects like hypotension and the baby was euglycaemic with no pre-feed or early morning hypoglycaemia on a combination of oral nifedipine and S/c octreotide. After extensive parent education, the baby was re-discharged with a CGMS probe in situ and CGM was done at home for the next few days. On follow-up, CGMS data showed good glycaemic control with interstitial fluid glucose levels never dropping below 40 mg/dL (vide Figure 1).

**Figure 2:**
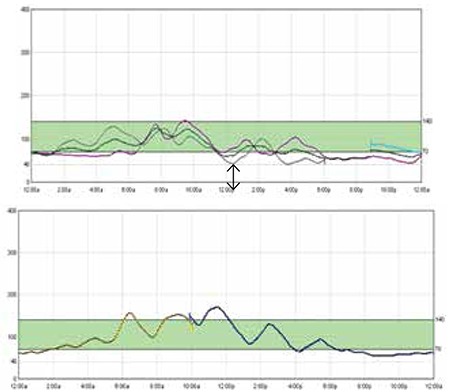
CGMS data at 11/2 year age showing hypoglycaemia on tapering off oral nifedipine with recovery on re-starting nifedipine in previous dosage.

After 4 months, S/c octreotide was gradually tapered off and stopped by one year of age. The child thereafter maintained euglycaemia on oral nifedipine only. Follow-up visits were difficult to arrange and at 11/2 years of age, the baby was admitted to determine if the dose of nifedipine also needed tapering. A CGMS monitor was inserted and oral nifedipine was tapered under supervision. However, following a hypoglycaemic episode, nifedipine was restarted in the previous dosage of 0.8 mg/kg/day. A high insulin level during the hypoglycaemic episode (insulin-19 μIU/mL when lab glucose was 29 mg/dL) confirmed the persistent hyperinsulinaemic state. CGMS data after readjusting nifedipine dosage to previous levels shows good glycaemic control (vide Figure 2).

## DISCUSSION

CHI can occur as a result of mutations in the subunits that form the KATP in pancreatic β-cells which modulates insulin secretion from the β-cells, namely the plasma membrane sulfonylurea receptor, ABCC8 and its associated inwardly rectifying potassium channel (KIR6.2) KCNJ11. Drugs which act on KATP channels, such as diazoxide, seem to need intact ABCC8 to be able to exert their effects (2). There is a keen interest to try and identify the exact genetic locus causing a particular abnormality in the β-cell in the hope that it may help in choosing the right therapeutic agent. However, mutation analysis of 13 patients with CHI of varying severity did not find mutations in the ABCC8 gene to be predictive of response to drugs (2).

Calcium-channel blockers, like nifedipine, have been reported to be effective in isolated case reports (7,8,9,10). Octreotide is a long-acting analogue of somatostatin, which has inhibitory effects on the release of insulin from pancreatic β cells. It has been successfully used in the short and long-term management of some patients with CHI in combination with frequent feeding (11). Another case has been reported to be successfully treated with a combination of nifedipine and octreotide (10). Glaser et al (12) reported a patient with CHI who was treated with octreotide and amlodipine (a calcium-channel blocker). It has been speculated that nifedipine, an L-type calcium-channel blocker and octreotide, which inhibits the Ca2+ entry into the pancreatic β-cells via voltage-operated Ca2+ channels of the L-type leading to suppression of insulin secretion (13), may have an additive effect (10).

The points of interest in our patient are: a) She was born of non-consanguineous marriage and had severe CHI inherited as autosomal recessive pattern; both parents being carriers. The maternally inherited p.R1419C mutation was detected by Sanger sequencing and has been reported previously (14). Dosage analysis of the ABCC8 gene by Multplex Ligation-Dependent Probe Amplification identified the paternally inherited heterozygous deletion of exon 3 (c.291-?_412+?del). The condition is rare in this population group in Asia where consanguineous marriages are uncommon. b) We were forced by circumstances to do a suboptimal pancreatectomy after which hypoglycemia persisted. Post-operatively, oral nifedipine was successful in controlling hypoglycaemia which remained uncontrolled by partial pancreatectomy and octreotide in a diazoxide-resistant case of CHI. Side-effects were not noted at our dose of 0.8 mg/kg/day. At present, at 11/2 yrs age, glycaemic control is maintained by oral nifedipine, while its withdrawal precipitates hypoglycemia. c) Use of nifedipine in adjunct to octreotide seemed to have an additive effect and it strengthens the speculation that calcium-channel blockers may work synergistically with octreotide, though the exact molecular mechanism is unclear. d) CGMS monitoring at home was found to be a feasible, safe option to monitor glycaemic control in a small baby. It reduced the need of frequent needle-pricks to monitor blood glucose and helped to allay parental anxiety regarding asymptomatic hypoglycaemic episodes.

Oral nifedipine was effective and well-tolerated at doses of 0.8 mg/kg/day in our patient. Hypotension is a potential side-effect (15) and has been reported at doses above 0.5 mg/kg/day (10). It seems that the therapeutic window of nifedipine is variable and hence needs individual customization and needs close monitoring during start of therapy. Short-acting nifedipine was cheap and easily available in soft gel capsules for adult use, but no liquid formulation was available. The capsulated short-acting form was known to be uns, photosensitive and heat-labile and had to be removed from the capsule and made up to proper strength before each dose.

Though the manufacturer does not recommend use of the CGMS to diagnose hypoglycemia, it has been used previously to detect hypoglycaemia and help titrate the dextrose concentration and other drugs (16,17). 24-hour trends have helped in better timing of medications so as to minimize the number of asymptomatic hypoglycaemic episodes. In our patient, the post-surgery CGMS data helped in documentation of euglycemia following the initiation of nifedipine therapy in adjunct to S/c octreotide and was particularly helpful in reassuring the parents on follow-up. We used CGMS data again at 11/2 years of age to document persistent hyperinsulinaemic state as well as good glycaemic control with oral nifedipine. In younger populations in whom the symptoms of hypoglycaemia are difficult to recognize and can be potentially life-threatening, CGMS can be an option to identify hypoglycemic episodes instead of frequent finger prick measurements. However, for us, a limiting factor was the cost of the device. Also as the sensor becomes old, there is increasing mismatch between the values of finger prick method (CBG) and CGM values. Though according to a meta-analysis (18), the sensor can give relatively accurate results for up to seven days; in our case, CGMS correlated well with CBG values till 4 days of insertion.

In conclusion, our case of diazoxide-resistant hyperinsulinism due to compound heterozygous ABCC8 mutations showed a good response to a combined treatment of nifedipine and octreotide. Further studies are required to evaluate the nifedipine effect and its dose range in patients with CHI.
